# Treatment of Fistula in Ano

**Published:** 1848-05

**Authors:** 


*Treatment of Fistula in Ano.*—The innovation which M. Huguier proposes in the performance of this operation consists in the suppression of the wooden gorget usually employed, and the substitution of a speculum ani in its stead. After the incision of the rectum the operator, guided by ocular inspection, can with facility investigate the condition of the parts. If the intestine is detached above the fistular orifice, it becomes easy to divide it with a pair of scissors; if several fistular passages exist, the speculum permits their easy detection, and their incision can be performed with far greater readiness than in the operation hitherto adopted. Indeed the advantages obtained by the use of the speculum in this surgical process are so evident that it is needless to insist upon the value of M. Huguier's discovery. The speculum should be formed of two valves, hinged to each other on one of their edges, and free on the other: a very simple mechanism permits these two valves to separate, and their interval is directed towards the diseased part of the rectum in such a manner as to expose it completely to view, and to render it tense and more easy to divide with the knife. Four cases have already been operated successfully by this method.—*London Medical Times.*

